# Driving Forces of Translocation Through Bacterial Translocon SecYEG

**DOI:** 10.1007/s00232-017-0012-9

**Published:** 2018-01-12

**Authors:** Denis G. Knyazev, Roland Kuttner, Mirjam Zimmermann, Ekaterina Sobakinskaya, Peter Pohl

**Affiliations:** 1 Johannes Kepler University Linz, Institute of Biophysics, Linz, Austria; 2 Johannes Kepler University Linz, Institute of Theoretical Physics, Linz, Austria

**Keywords:** SecY, Proton motive force, Translocation

## Abstract

This review focusses on the energetics of protein translocation via the Sec translocation machinery. First we complement structural data about SecYEG’s conformational rearrangements by insight obtained from functional assays. These include measurements of SecYEG permeability that allow assessment of channel gating by ligand binding and membrane voltage. Second we will discuss the power stroke and Brownian ratcheting models of substrate translocation and the role that the two models assign to the putative driving forces: (i) ATP (SecA) and GTP (ribosome) hydrolysis, (ii) interaction with accessory proteins, (iii) membrane partitioning and folding, (iv) proton motive force (PMF), and (v) entropic contributions. Our analysis underlines how important energized membranes are for unravelling the translocation mechanism in future experiments.

## Introduction

### Structural Insights into SecY Conformational States During Translocation

**Fig. 1:**
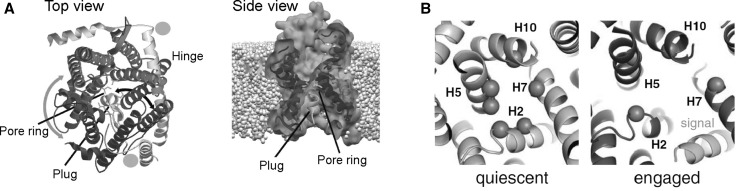
**a** Crystal structure of the *Methanococcus jannaschii* SecY channel in its resting state (van den Berg et al. 2004) (PDB 1RH5). Left panel: channel viewed from the cytosol. Right panel: channel side view from the plasma membrane. **b** Closer view on the hydrophobic seal formed by the pore ring residues (green spheres). The pore ring of *H. sapiens* Sec61α is formed by 3 Ile, 2 Lue, 1 Val (right panel); and of *M. jannaschii* SecY by 4 Ile, 1 Leu, 1 Val (left panel). Complex of Sec61 with a translocation intermediate (right panel: engaged structure in red, signal sequence in cyan, PDB 3JC2) (Voorhees und Hegde 2016) as compared to the resting state of SecY (left panel: quiescent structure in grey, PDB 1RH5). (Color figure online)

The Sec machinery is responsible for the reconstitution and translocation of many bacterial cytoplasmic, outer membrane and secretory proteins. The core element of the Sec translocation machinery is the heterotrimeric translocon SecYEG which resides in the cytoplasmic membrane (van den Berg et al. 2004). SecYEG has a striking homology with archaeal and eukaryotic analogues SecYEβ and Sec61αβγ (Bondar et al. 2010). All Sec translocons have a central pore that is closed for the passage of molecules in its resting state, due to the ring of hydrophobic amino acids (marked green in Fig. 1a) and the re-entrant loop TM2a also called the plug domain (marked yellow) (Saparov et al. 2007). The main translocation unit of the translocon SecY can be seen as a clamshell (blue and red halves) with a hinge region clamped by SecE, and the so-called lateral gate—composed of transmembrane helixes TM2b and TM7 on opposite sides (van den Berg et al. 2004). It was suggested that conformational transitions between the open and closed states can be seen as a rigid body movement of one-half against the other (Park et al. 2013).

Bacterial proteins can either be translocated via SecYEG, when still being translated by the ribosome (co-translationally) or after having been fully synthesized (post-translationally). Transmembrane proteins usually take the co-translational pathway where the protein is inserted into the lipid bilayer by the complex of the translating ribosome and the translocon. The secretory or outer membrane proteins that still contain their signal sequences (pre-proteins) use the post-translational pathway which requires a complex of SecY with the motor protein SecA (Junne et al. 2007; Denks et al. 2014). SecA was reported to interact with the substrate as it emerges from the ribosome (Huber et al. 2011). Co- and post-translational processes might even be intertwined, since SecA and the ribosome have overlapping binding sites on SecY and hence compete for binding (Kuhn et al. 2011).

To gain insight into how the translocon accommodates its substrates during translocation, we collected key structural information (Table 1). The structures can be grouped into those that show the resting (SecY alone), primed (with translocation partner bound such as SecA or empty ribosome), and engaged (in complex with translocation substrate) states of SecY. Since SecY engaged in translocation has only been captured in low resolution (except for the structure 5EUL (Li et al. 2016) mentioned below), Table 1 contains a mix of high- and low-resolution structures. We also included the RNC-Sec61 complex for comparison [pdb 3J46 (Voorhees et al. 2014)]. This structure is especially valuable because it captures the translocation intermediate without artificial cross-linking of the substrate to the translocon. SecY’s constriction zones regulate protein translocation. They consist of the hydrophobic ring for secretory proteins, and the lateral gate for transmembrane proteins. In addition to the width of these two important regulatory elements, the position of the plug domain—the third regulatory element—is of interest, because it governs the translocon’s permeability (Saparov et al. 2007). Table 1 summarizes current knowledge about all three regulatory elements:

(i) In the RNC-Sec61, the plug has not been resolved, conceivably because it is mobile during translocation. To highlight plug mobility, Table 1 reports plug distances from two different reference points (see Table 1).
(ii) The hydrophobic ring adopts the form of an ellipse with one axis being about twice as large as the other. It transforms into a more circular shape when the translocon is primed or engaged.
(iii) Lateral gate opening tends to be minimal in the resting state. Different organisms such as *M. jannaschii* and *T. thermophilus* are almost identical in terms of lateral gate widths and “ring” shapes and sizes.
(iv) The engaged structures either have a resolution which does not allow us to reliably distinguish between the conformational transitions in the constriction zone, or actually reproduce the resting state with astonishing accuracy, probably because the SecYEG dimer is a poor model of the engaged SecY (pdbs 5CH4 for engaged vs. 5AWW (Tanaka et al. 2015) and 2ZJS (Tsukazaki et al. 2008) for resting states). Another engaged structure proposes even greater squeezing of the “ring” when compared to the ellipse of the resting state, with simultaneous tightening of the lateral gate (pdb 5GAE (Jomaa et al. 2016)). How (a) substrate sampling between the aqueous environment of the channel and the hydrophobic membrane interior or (b) the translocation of co-translational substrates may occur under these conditions has remained unclear. The structures 3MP7 (Egea und Stroud 2010) and 5EUL (Li et al. 2016) are of high resolution and do show the expected ring widening and lateral gate opening. However, the former structure is that of the SecY dimer which raises the question of its relevance to the physiological event of protein translocation that now is believed to be conducted by the SecYEG monomer. The latter structure includes artificial cross-linking and a SecA-substrate fusion construct which may have biased the conformation.

**Table 1:**
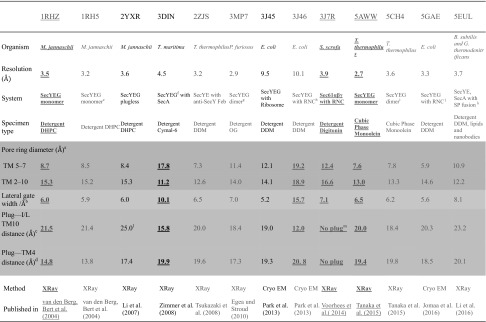
Comparison of high- and low-resolution structures of SecY. (Color table online)

In addition, one Sec61 structure is included for comparison. The functional state of the ring (violet box), lateral gate (tan box) and plug (orange box) can be assessed from the indicated distances. Structural data (i) in the absence of a SecY ligand (red), i.e. of the idle SecY monomer, are compared to (ii) those of SecY bound to an empty ribosome or to SecA (black) and (iii) those captured with a translocation intermediate (blue). The structures that best represent these three states are highlighted (underlined and bold)

^a^Pore ring diameter: between centres of mass of ILE of TM2 and TM10, TM5 and TM7

^b^Lateral gate minimal width: minimal distance between TM2 and TM7 backbone Cα (or C) atoms

^c^Plug—I/L TM10 distance: between centre of mass of Plug and centre of mass of ILE (or LEU) residue of the TM10, sort of "z" coordinate (along the normal to the bilayer) of the centre of mass of plug

^d^Plug—TM4 distance: between centre of mass of Plug and centre of mass of TM4, sort of "x" (or "y") coordinate of the centre of mass of plug

^e^Mutation: K422R, V423T for more stable structure

^f^SecA (1-816) in the presence of ADP

^g^C-terminal SecY loops protrude into channels in a quasi dimeric state

^h^Cross-link between SecYEG and a Ribosome Nascent Chain (DsbA signal peptide with Cysteine at pos. 19 and SecM arrest peptide; total length 100aa)

^i^SecE protrudes into lateral gate of 2nd SecY

^j^Ribosome–Nascent Chain (artificial signal peptide; phoA and SecM arrest peptide) complex with SRP and SR

^k^Crosslink between SecA-OAins and SecY: SecA-OAins derived from *B. subtilis* with 49 amino acids of *E. coli* OmpA including the signal peptide, inserted into the SecA 2-helix finger. SecYE from *G. thermodenitrificans* with amino acids 202–213 replaced with TFGGLN and AYC08 from *V. pacos* as nanobody assisting in crystallization

^l^The plug in our calculations is defined as a segment between helixes 1 and 2b. Hence the plug deletion mutant of SecY still has an assigned distance here, because the new pseudo-plug is formed, as was shown for SecY from *M. jannaschii* with the deletion of residues (57–67), where the new loop was formed from residues (55–56) and (68–71) (Li et al. 2007)

^m^No number is assigned, as the plug was not visible in the structure

Structural investigations are invaluable for understanding the intermolecular rearrangements during translocation. However, their interpretation is limited by several factors: First, stable translocation intermediates as stalled RNC (ribosome–nascent chain complexes) or proOmpA-SecA fusion products are all cross-linked to SecY [except for PDB 3J7R (Voorhees et al. 2014)]. Of course, cross-linking proves that the contact can take place, but it does not confirm its statistical relevance. Thus, in the absence of translocation dynamics we are lacking information as to whether the pdb snapshot reflects the prevalent translocon conformation during translocation.

Second, all high-resolution structures of SecY have been obtained either in detergent or in the cubic phase of monoacylglycerol monoolein. Both environments differ in terms of hydrophobic thickness, intrinsic curvature, surface charge, and pressure profile from the *E. coli* plasma membrane. Stretching forces at the lipid bilayer–water interfaces, compression in the membrane midplane (Cantor 1999) as well as electrostatic interactions or hydrophobic mismatch may well shift the equilibrium between the closed and open states. An example is provided by the interaction between negatively charged lipids and the N-terminus of SecA (Bauer et al. 2014). It forces SecA to be in a different conformation (Koch et al. 2016) than captured for the SecA-SecY structure in detergent (Zimmer et al. 2008).

**Fig. 2:**
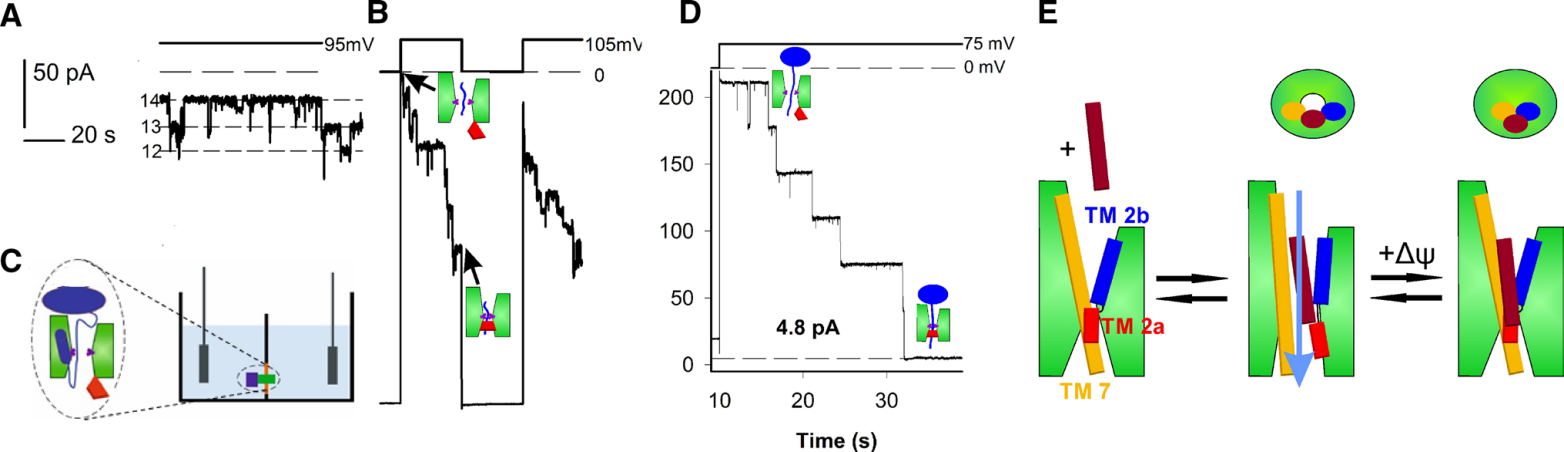
Electrophysiological single channel measurements of SecYEG, purified and reconstituted into planar bilayers. **a** Single channel activity appeared only after addition of the signal peptide SP (N-terminal 20 residues from the pre-protein proOmpA). Numbers on the left correspond to the number of single channels, the distance between the two adjacent dashed lines corresponds to the current through a single channel. **b** An increase in transmembrane potential led to a decrease in membrane conductivity in a series of steps. Each conductivity step corresponds to the closure of a single channel. Conductivity almost fully recovered when no transmembrane potential was applied, indicating that SP remained bound to the lateral gate even in the closed state of SecY. **c** Experimental scheme for single SecYEG (green) channel measurements. The transmembrane potential was controlled via two AgCl-electrodes (grey) that were also used for monitoring bilayer (orange) conductivity. The bound proOmpA-DHFR is in blue. **d** Voltage-gated closing of SecYEG was also observed when the fusion protein proOmpA-DHFR was added instead of SP. The residual leakage is given in pA. **e** Putative scheme of voltage-driven channel closure. The resting channel is closed (left). SP binding opens the translocon (middle). It becomes permeable to the translocating peptide chain as well as to ions and water (represented by blue arrow) in the absence of physiological values of the transmembrane potential Δψ, which would close the channel (right). The stalled translocation intermediate is likely to be pushed from the lateral gate into the lipid. The upper panel shows the channel from the cytoplasm. TM stands for transmembrane helix. Single channel amplitude for A, B, D was 0.7 ± 0.1 pS/mM. Δψ is shown on the top. The small insets depict SecYEG (green) with a translocation intermediate (blue), SecYEG’s plug (red), and two of hydrophobic ring’s Ile (violet). The figure is taken from Knyazev et al. (2014). (Color figure online)

Third, all of these structures lack transmembrane potential, to which SecY (but not Sec61) is exposed. In contrast to most of the structures with a translocation intermediate such as 3J46 (Park et al. 2013), 5GAE (Jomaa et al. 2016), and 5EUL (Li et al. 2016), the reconstituted SecY-signal peptide, SecY-proOmpA complex, or SecY-ribosome complex appear to be ion permeable at small transmembrane potentials (Knyazev et al. 2013; Knyazev et al. 2014). The complexes exclude ions when physiological values of membrane potential (Fig. 2b, d) are applied indicating that under physiological conditions, the conformation of the translocation intermediate is different from published structures (Knyazev et al. 2014). Assigning the functional properties of translocons at physiological temperatures to structural data obtained from translocons trapped in stable, low-energy conformations by cryo-EM or by X-ray crystallography is everything but straightforward. The potential problem of relating structural data obtained from low-energy conformations and functional data obtained at physiological temperatures is nothing peculiar to translocons, but it could be more important than it is for many other membrane proteins. For example, there has been excellent concordance between structural and functional data for K^+^ channels (Bezanilla 2008; Vargas et al. 2012). But, these channels have a rigid selectivity filter and relatively fixed conformations of inner and outer vestibules, with the exception of movements associated with gating. In contrast, it is clear from functional studies that the translocon has a very dynamic range of conformational states that can accommodate a large variety of protein structures during translocation. Therefore, it is very possible that the gating properties of the translocon could be dependent on structural rearrangements that will never appear in the stable structures obtained by cryo-EM or X-ray-crystallography. The importance of this point is underscored by the strong temperature dependence of the opening of the Sec61 translocon. Although sealing of the Sec61 pore by BiP was widely accepted, this sealing was electrophysiologically only observed at 5 °C (Wonderlin 2009), but the pore reopened with warming to physiological temperatures. From a biophysical point of view, the difference in temperature at which structural and functional data are collected is a potentially important stumbling block in reconciling different models for the structural basis for the functional properties of translocons.

### Maintenance of the Membrane Barrier to Small Molecules by SecY Engaged in Translocation as Reported by Electrophysiological and Other Functional Assays

Because the translocon is even closed to water in its resting state (Saparov et al. 2007), protein translocation requires a conformational transition to an open state. The lumen of the open state may be as large as 2.4 nm in diameter as has been observed with substrates that harboured rigid organic tails of different sizes (Bonardi et al. 2011). Such a large opening offers the possibility of translocating α helices in their folded state. In contrast, β-sheets have to pass in an unfolded state, since β barrels are even larger. Unfortunately, the resolution of available structures in the pdb databank is too low to unambiguously discern the secondary structure of α helical polypeptides in the SecY lumen (Table 1). However, there is little doubt that the signal sequence of pre-proteins is folded when bound in the lateral gate region (Fig. 1b). It was reported that folded α helical substrates are translocation-competent (Lecker et al. 1990), but they still might be unfolded during translocation. Unfolding may not be complete as successful translocation of proOmpA with an intramolecular loop formed by cross-linked cysteines was reported (Tani und Mizushima 1991).

Electrophysiological measurements of the purified and reconstituted SecYEG complex show SecYEG openings by pre-proteins or isolated signal peptides (Knyazev et al. 2014) (Fig. 2). Pore size estimates of ~ 0.4 nm from these measurements agree very well with those obtained by ion conductivity measurements of plug deletion mutants (Saparov et al. 2007). Such a diameter is large enough to allow unfolded peptide chains to be accommodated in the aqueous pore. It is truly surprising that pore size does not seem to depend on the presence of a peptide in the lateral gate (Knyazev et al. 2014), indicating that the translocation must possess unusual plasticity.

Ribosomes are also capable of opening the SecY channel (Knyazev et al. 2013), even though their affinity to the translocon seems to be smaller than that of pre-proteins. The same phenomenon has been reported for the eukaryotic translocon Sec61 (Erdmann et al. 2010), which may well result in an open state of the idle translocon at physiological temperatures (Wonderlin 2009). The resulting ion leak through the membrane of the endoplasmic reticulum was reported to be somehow inhibited by a calcium-dependent interaction of calmodulin with Sec61α (Erdmann et al. 2011).

Both prokaryotic and eukaryotic translocons appear to be voltage-gated. In both cases the probability that the channel was in the closed state increased with the absolute value of transmembrane potential (Knyazev et al. 2014; Wonderlin 2009; Erdmann et al. 2010). Since the membrane of the endoplasmic reticulum is thought to be de-energized, i.e. to possess no significant transmembrane potential, Sec61’s voltage dependence must be an evolutionary relict that the eukaryotic translocon inherited from its bacterial and archaeal ancestors. In contrast, voltage gating of SecY appears to be crucial for maintaining the membrane barrier to small cations and protons (Knyazev et al. 2014). The failure to do so would destroy the proton gradient across the bacterial plasma membrane, and thus would be lethal. The symmetric voltage dependence of translocon’s opening suggests that mobile sensor domains may not be the gating elements. Instead, voltage-driven bilayer thinning may gate the translocon. If so, electrostriction would induce a mismatch between the hydrophobic thicknesses of protein and membrane core. Mechanosensitive channels are known to respond to such a mismatch by a conformational change that eventually leads to channel opening (Perozo et al. 2002). Commonly a mechanical tension is applied that reduces membrane thickness by 2–5% (Sachs 2015). In contrast, electrostriction is less efficient. The bilayer thickness decreases with a factor $2 \times {10^{ - 2}}/{V^2}$ (Alvarez und Latorre 1978). That is, for a transmembrane potential of Δψ ~ 100 mV we find $\frac{{\Delta C}}{C}=\frac{{\Delta d}}{d}=\alpha {V^2}=2 \times {10^{ - 4}},$ or 0.02%, where *C* and *d* denote capacitance and thickness of the bilayer, respectively. Since hundredfold smaller that reported for the gating of mechanosensitive channels, this moderate thinning must be negligible. Otherwise, tiny temperature fluctuations would govern the translocon. This rules out electrostriction as a possible mechanism for gating the translocon.

Circumstantial evidence suggested that cation exclusion by SecY would help preserve the proton motive force (Dalal und Duong 2009). However, direct electrophysiological measurement revealed that SecY from *E. coli* prefers anions over cations by only about sevenfold (Sachelaru et al. 2017). The anionic selectivity of mammalian and yeast translocons from *C. familiaris* and *S. cerevisiae* is even smaller, with the permeability for anions being only about 1.5 times higher than for cations (Erdmann et al. 2010).

### Role of the Plug

Interaction of the plug with the pore-forming helices is thought to stabilize the closed state of the SeYEG channel (Li et al. 2007). Accordingly, plug relocation from the constriction zone is likely to lead to lateral gate opening and widening of the hydrophobic ring. This is in line with cross-linking experiments in which immobilizing the plug in the vicinity of the SecE subunit (a forced open state) by a disulfide bridge renders SecY permeable to ions (Saparov et al. 2007). Disulfide bridge formation between these two rather distant elements in the crystal structure indicates that the plug must be rather flexible. Indeed, cross-linking of overexpressed SecY from inner membrane vesicles (IMVs) showed that plug movement can be restricted without abrogation of translocation. Therefore, the linker between the plug and helix 10 must be at least 8 Å in length (Lycklama a Nijeholt et al. 2010).

Contrary to the idea that plug dislocation is required for the opening of the lateral gate, the hydration of fluorescent labels on different positions on plug did not change upon insertion of co-translational translocation substrates (Lycklama a Nijeholt et al. 2010, 2011). Yet plug movement is accompanied by changes in the hydrophobicity of its environment as has been observed when the translocation intermediate consists of a post-translational substrate. To reconcile this observation with the ion channel activity that we see under comparable conditions for similarly de-energized reconstituted bilayers (Sachelaru et al. 2017), we have to assume that channel opening is not associated with plug movement but due to a widening of the pore ring.

### Openings of the Lateral Gate

The opening of the lateral gate is essential for translocation. This follows from experiments with SecYEG containing IMVs, where bis-maleimides of different lengths were cross-linked to both poles of the lateral gate. Long linkers allowed translocation, whereas the shorter ones abrogated it (du Plessis et al. 2009).

Blocking both the eukaryotic Sec61α and the prokaryotic SecY in vivo by decatransin and cotransin (Junne et al. 2015; du Plessis et al. 2009) is in line with this conclusion. These blockers presumably bind to the constriction zone of the lateral gate in the closed state of the translocon. This way intercalation of the incoming signal peptide is prevented. Abrogating the tight ion seal of the closed translocon by introducing *prl* mutations (Saparov et al. 2007), also weakened inhibitor binding. This observation suggests that electrophysiological experiments on planar bilayers should be able to discern a smaller opening probability of the cotransin pre-treated translocon in the presence of pre-proteins.

### ATP or GTP Hydrolysis as a Driving Force of Translocation

*Co-translational translocation* involves ribosome binding to the translocon and translocation is initiated while the elongation process of the translation is underway. The ribosome–SecY interaction interface is conserved and includes cytoplasmic loop 6/7 from the SecY side and ribosomal protein ul29 in prokaryotes (Prinz et al. 2000; Jomaa et al. 2016). The signal recognition particle (SRP) binds to a hydrophobic stretch of a signal sequence or transmembrane helix and helps to guide the nascent chain to the translocon with the help of the SRP-receptor (SR) in both eukaryotes and prokaryotes [for details see the review (Saraogi und Shan 2011)]. SRP-SR driven insertion of the first hydrophobic segment of the nascent chain into the translocon is thought to be GTP-dependent (Ataide et al. 2011). However, further translocation does not require SRP-SR (Miller und Walter 1993) and is conventionally assumed to be driven by the translational elongation, i.e. also GTP-driven. However, a polypeptide loop between the ribosome exit tunnel and the translocon [pdb 3J46, (Park et al. 2013)] rules out direct peptide pushing by the ribosome.

This is in line with the requirement for SecA to translocate the membrane protein RodZ, which has a 200 residues-long periplasmic domain (Wang et al. 2017). Obviously, bacterial ribosomal chain elongation *per se* does not provide a driving force for translocation of large cytoplasmic loops. It is worth noting that ribosomal chain elongation can still be rate limiting as alternative codons may lead to a threefold faster translocation (Sørensen und Pedersen 1991). Accordingly, by reducing the overall translational speed, the elongation inhibitor cycloheximide affects translocation, which in turn modifies the topology of membrane proteins (Goder und Spiess 2003).

In addition to GTP, the hydrophobic effect may fuel translocation: Once the elongating chain is hydrophobic enough, the free energy difference between its non-inserted and inserted states drives the chain into the lipid phase. The gain in energy may be large enough to overcome translation arrest (Ismail et al. 2012). For more hydrophilic substrates, the proton motive force may gain importance as an energy source for translocation. It has been shown to accelerate co-translational translocation (Ismail et al. 2015).

*Post-translational translocation* was proposed to be driven by the ATPase SecA (Hartl et al. 1990). Structural and biochemical research was applied to gain a more general understanding about the role of SecA homo-dimerization and binding kinetics to lipids and SecY. Under physiological conditions, SecA is predominantly a dimer with a cellular concentration of about 5 µM (Woodbury et al. 2002; Auclair et al. 2013). Within the translocon complex and upon lipid binding, SecA may act as a monomer (Or et al. 2005) or as a dimer (Kusters et al. 2011). That is, there is no consensus about the oligomeric state during translocation (Allen et al. 2016). The 20 N-terminal residues of SecA are important for binding to SecYEG and are also required for SecA-lipid interactions (Floyd et al. 2014; Bauer et al. 2014; Gouridis et al. 2013; Hendrick und Wickner 1991). Binding also depends on the nucleotide in SecA’s binding pocket: AMP-PNP, the non-hydrolysable analogue of ATP, augments SecA’s affinity to SecY by three orders of magnitude as compared to ADP (Deville et al. 2011).

**Fig. 3:**
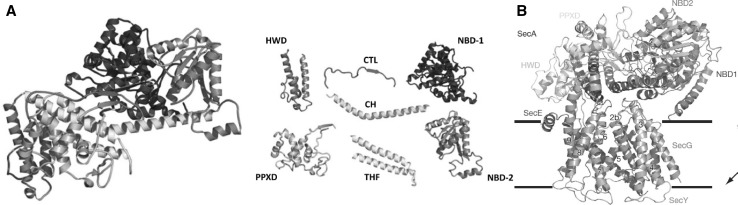
**a** Ribbon representation of the *B. Subtilis* SecA protein coloured by domain (Left) with the individual domains shown on the right (PDB 1M6N). They include (N-terminal to C-terminal on SecA): the nucleotide-binding domain-1 (NBD-1) (blue), the pre-protein cross-linking domain (PPXD) (gold), the nucleotide-binding domain-2 (NBD-2) (light blue), the central helix subdomain (CH) (green), the helical wing domain (HWD) (dark green), the two-helix finger subdomain (THF) (cyan), and the carboxyl-terminal linker (CTL). The CTL is depicted in red and serves as a model of PhoA signal peptide. From Zhang et al. (2017). **b** Cartoon of the SecA-SecYEG complex viewed from the side. Subunits of SecYEG and domains of SecA are subscribed according to colour code. The lines indicate the membrane boundaries. The two-helix finger of SecA inside the cytoplasmic funnel of SecY. From Zimmer et al. (2008). (Color figure online)

Binding of the SecA-ATP complex may widen the SecY lumen while leaving the plug in a pore-sealing position (Zimmer et al. 2008; Allen et al. 2016) (Fig. 3b, pdb 3DIN in Table 1). However, the 3DIN structure has been captured in detergent and, thus, SecA’s amphipathic N-terminus adopts a position that is incompatible with its membrane interaction outlined above. For this interaction to occur, a major conformational change involving a 30 Å translational movement is required (Koch et al. 2016), which could potentially impact SecY’s plug position.

A scenario in which SecA regulates the state of SecY via ATP hydrolysis (Liang et al. 2009) is compatible with a mechanism in which SecA allows forward diffusion of the substrate, but blocks its backsliding. This hypothesis was also substantiated by single-molecule FRET experiments and molecular dynamics simulations (Allen et al. 2016). It is usually called Brownian ratcheting.

An alternative hypothesis of SecA-driven translocation envisions ATP hydrolysis to fuel conformational changes of SecA that result in an active pushing of the substrate. This power stroke hypothesis assigns the role of the pushing element to the two-helix finger (THF) of SecA (Zimmer et al. 2008; Bauer und Rapoport 2009) (Fig. 3a). SecA would push the bulky residues of the substrate, allowing the smaller ones to partially slide back (Bauer et al. 2014). This would allow the translocation of secretory proteins independent of their sequence—especially if the power stroke is coordinated with peptide binding and release from SecA’s pre-protein binding domain (PPXD).

However, it remains enigmatic how ATP hydrolysis in the NBD1 domain (Economou et al. 1995) translates into the big conformational change on the opposite side of the molecule, the THF domain, which is at the C-terminal part of the helical scaffold domain, HSD (for domain structure see (Hunt et al. 2002) and Fig. 3a). Thus, THF may have an alternative function. It may prime translocation by inserting the hairpin formed by the substrate’s signal peptide and the following segment (Zhang et al. 2017).

Translocation kinetics was explored in order to distinguish which model describes SecA-driven translocation best: power stroke or Brownian ratchet. The power stroke hypothesis for SecA was reported to be in line with the linear dependence of translocation time (τ) on the substrate length (*N*) in the absence of PMF (Tomkiewicz et al. 2006). Interestingly, such linear dependence *τ*(*N*) was subsequently reproduced in the presence of PMF but not in its absence (Liang et al. 2009) (Fig. 5b). This observation was interpreted in terms of a Brownian ratcheting mechanism in which SecA only functioned to regulate pore accessibility.

The dependency *τ*(*N*) was extensively researched for completely different translocation systems. For example, the translocation time of double-stranded DNA through solid state nanopores (made in alumina-coated silicon nitride) was found to be proportional to DNA length (Chen et al. 2004). The same was reported for single-stranded DNA in experiments with alpha hemolysin (αHL) pores (Kasianowicz et al. 1996). To ensure the polymer’s constant translocation velocity, the dragging force (here electrophoretic, acting on the negatively charged polymer when voltage was applied across the pore) must have been counterbalanced with the viscous force from the polymer–pore interaction.

In contrast to single- or double-stranded DNA that move through rigid pores, SecA-driven polypeptides may unfold on the cytoplasmic side or fold on the periplasmic side, thus delaying or accelerating translocation. Differences in the interaction of SecY’s translocation pore with various amino acid side chains only exacerbate the deviations from linearity. Such deviations were even found for the seemingly much more homogeneous DNA when passing through solid state nanopores (Storm et al. 2005). Thus, attributing a linear dependence *τ*(*N*) either to a power stroke or a Brownian ratcheting model does not appear to be straightforward.

### Accessory Proteins May Drive Translocation

**Fig. 4:**
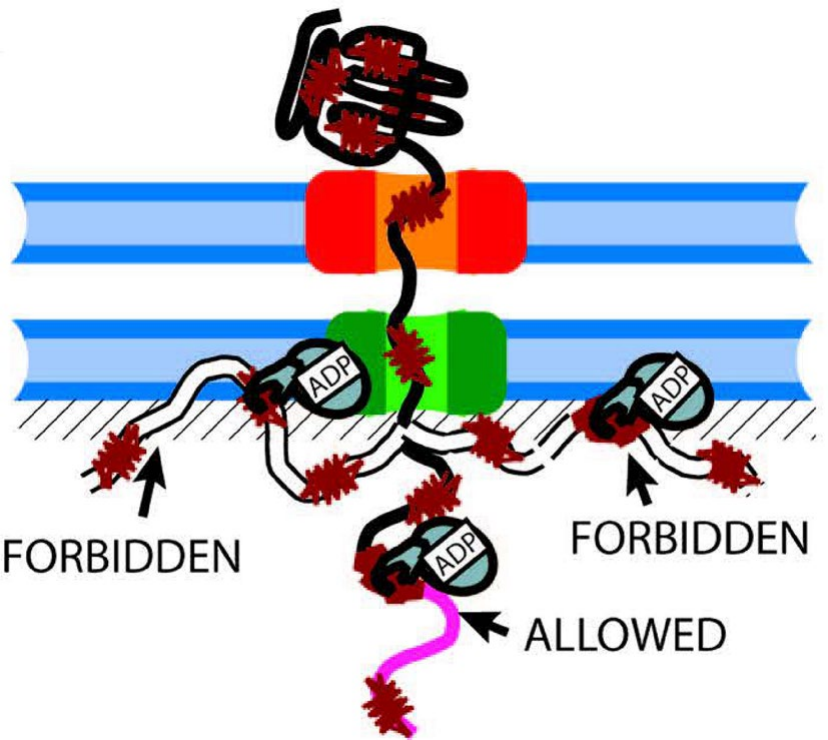
Schematic view of translocation events that are driven by polypeptide folding (direction of translocation is from top to bottom). The number of possible conformations (in pink) without mtHsp70 is much larger than in its presence. That is, mtHsp70 binding to a translocating polypeptide reduces the number of “allowed” polypeptide conformations, i.e. conformations within the mitochondrial matrix space. It increases “forbidden” conformations (in white) due to additional spatial constraints. As a result, the accelerated folding process may exert a pulling force on the polypeptide within the translocon. From De Los Rios et al. (2006). (Color figure online)

For a detailed review of SecY’s and Sec61’s translocation partners, the reader is referred elsewhere (Veenendaal et al. 2004; Park und Rapoport 2012; Lang et al. 2017). Here we only briefly discuss the topic from the perspective of accessory proteins as driving forces for translocation. Periplasmic chaperones such as Skp (Schäfer et al. 1999) or PpiD (Antonoaea et al. 2008) were reported to facilitate translocation via binding to the peptide chain which emerges from the periplasmic side. Thus, they prevent the polypeptide chain from backsliding, thereby contributing to the previously mentioned Brownian ratchet mechanism. Conceivably, the mitochondrial Hsp70 family chaperones operate according to a similar mechanism (Simon et al. 1992). In addition, to favour unidirectional diffusion, chaperones also assist in polypeptide folding. The resulting entropic pulling forces were reported to be above 10 pN (De Los Rios et al. 2006). The pulling comes with a penalty, due to a decrease in the chain’s entropy. This penalty diminishes with an increase in the total number of bound residues (Fig. 4).

Cross-linking experiments and co-purification of additional components alongside of SecYEG suggest there might be additional translocation interaction partners (Boy und Koch 2009; Sachelaru et al. 2014; Sachelaru et al. 2013). Of those, we will point out the proton channel SecDF which was reported as a possible component which couples the pH gradient and translocation (Arkowitz und Wickner 1994; Tsukazaki et al. 2011). In marine bacteria, the SecDF paralog exists and is suggested to employ the gradient of Na^+^ instead of H^+^ (Ishii et al. 2015). It was speculated that SecDF assists translocation by (i) first allowing its periplasmic P1 domain to bind to the emerging peptide chain, and (ii) second performing a conformational change that moves P1—along with the bound amino acids of the chain—away from the pore. SecDF is thought to use the proton gradient for both the conformation switch and binding-release cycles (Tsukazaki et al. 2011). This mechanism was supported by MD simulations (Ficici et al. 2017), which concluded that the P1 head movement is regulated by the transmembrane potential. The barrier for such transition is lowered in the presence of the transmembrane potential Δψ. The barrier for the reverse transition is lowered when Δψ is absent. Δψ oscillates along with SecDF conductivity, because proton flow along the existing pH gradient acts to reduce Δψ. This implies that substrate binding regulates SecDF’s open probability, and that SecDF is proton selective. The reported single channel amplitude of about 100 pS in neutral pH (Tsukazaki et al. 2011) is at odds with this anticipation. Since proton selective channels generally possess a tiny unitary conductivity that is too small to be resolved in single channel recordings (Decoursey 2003), the observation suggests that SecDF conducts other ions as well. SecDF is absent from Crenarchaea and organisms belonging to the thermoplasma group (at least from those which have been fully sequenced) (Bolhuis 2004). Many of those organisms are acidophiles, which might indicate that SecDF is not required in case of large transmembrane pH gradients.

**Fig. 5:**
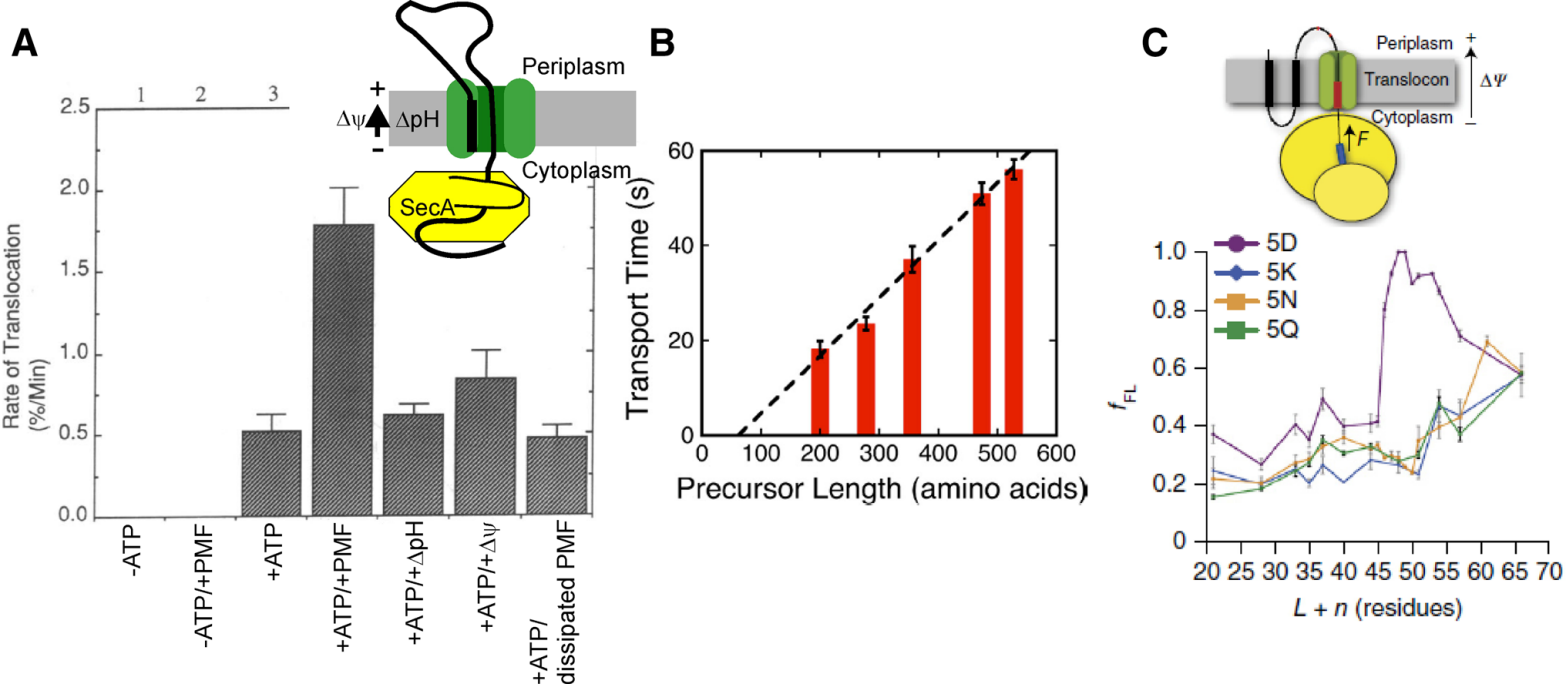
**a** Rate of translocation is stimulated by PMF (Eq. 1). *Δψ* and ΔpH are interchangeable. The figure is taken from Driessen und Wickner (1991). **b** In the presence of PMF, the time required for translocation (= transport time) increases linearly with the length of the polypeptide (= precursor length) that is translocated. The figure is taken from Liang et al. (2009). **c** PMF acts by exerting an electric pulling force on nascent chains containing negatively charged residues (located in the region marked in red). The force is sufficient to trigger the release from translation arrest (arrest peptide in blue) that has been imposed by SecM. f_FL_ stands for the fraction of the peptide that has been translocated. The x axis shows the distance from the arrest peptide to the charged stretch (red). f_FL_ was largest when the red segment contained acidic residues (5D). However, it also increased in case of basic (5K) and non-charged (5N, 5Q) residues. Simple pulling by *Δψ* cannot explain this observation. The panel is taken from Ismail et al. (2015). The colour schemes on top describe the experimental system used: in vitro post-translational translocation for A–B and co-translational translocation for C. (Color figure online)

It is worth mentioning that the minimal translocation machinery containing SecYEG is also capable of coupling *Δψ* to peptide translocation—even though with lower efficiency than the larger complex including SecDF (Schulze et al. 2014). A number of experiments lacking SecDF (Driessen und Wickner 1991; Liang et al. 2009) show a *Δψ* -mediated acceleration of the translocation (Fig. 5a, b). Thus, SecDF does not appear to be essential for translocation, but it may facilitate translocation by a yet unknown mechanism.

### PMF-Driven Translocation

SecYEG resides in a permanently energized membrane—the cell sustains the electrochemical gradient of protons across the cytoplasmic membrane, also known as PMF. This energy might be utilized by the translocation system, although probably not by the eukaryotic one, as no significant PMF is built across the membrane of the endoplasmic reticulum.

PMF consists of electrical (transmembrane potential *Δψ*) and chemical (pH gradient) components:

1 $${\text{PMF}}=\Delta \psi - 2.3\frac{{RT}}{F}\Delta {\text{pH}},$$

where *R* and *T* represent the gas constant and absolute temperature, respectively.

The first observation of the translocation rate increasing effect of the electrochemical gradient of protons was made on inner membrane vesicles containing SecYE (Driessen und Wickner 1991) (Fig. 5a). The proton–potassium antiporter nigericin was used to dissipate ΔpH, the potassium ionophore valinomycin—to dissipate *Δψ*. The changes in inner pH and transmembrane potential were monitored with fluorescent sensors pyranine and Oxonol VI. PMF seemed to accelerate transport, even though *Δψ* across the bilayer of IMVs was smaller than in *E. coli* cells: judging from Oxonol VI quenching (~ 10%), the absolute value of *Δψ* was only about 10 mV. This estimate is much smaller than what is usually expected for IMVs: *Δψ* ~ 80 mV (Keyzer 2002). The reason for the discrepancy is unclear. Proton pumping and proton consumption by SecY may have anyhow resulted in a non-uniform *Δψ* distribution along the IMV bilayer. Subsequent measurements with a fluorescence translocation assay (Liang et al. 2009) also showed that PMF increases the translocation rate. Moreover, complete translocation of proOmpA with an intramolecular crosslink between residues 290 and 302 was only observed in the presence of PMF, suggesting that PMF is essential for the translocation of larger substrates (Tani und Mizushima 1991).

How does each of the two *PMF* components facilitate translocation? The SecM stalling sequence was used in vivo as a force probe to estimate the pulling force that *Δψ* exerts on the nascent chain during translocation (Fig. 5c) (Ismail et al. 2015). *Δψ* stimulates movement of acidic residues through the translocon, thereby releasing the translation arrest of the nascent chain. Conceivably, *Δψ* was not the sole driving force as segments enriched in basic residues were also freed from the arrest.

*Δψ* does not impose the “positive inside” rule by retarding the passage of positive charges, i.e. it is not responsible for the topology of membrane proteins. Otherwise the reversal of *Δψ* in acidophiles would have led to an enrichment of the periplasmic leaflet with the positively charged amino acid residues arginine and lysine. Rather anionic lipids act to anchor the positive charges to the cytosolic leaflet enabling acidophiles to follow the positive inside rule (van de Vossenberg et al. 1998).

According to (Zilberstein et al. 1979) PMF in living *E. coli* cell amounts to 150–200 mV. With *Δψ* ranging from 100 to 150 mV (negative in cytoplasm), trivial electrostatic considerations give a pulling force that acts on a single elementary negative charge of about:

2 $$qE=1.6 \times {10^{ - 19}}\,{\text{C}} \times \frac{{0.1\,{\text{V}}}}{{{{10}^{ - 9}}{\text{m}}}}\sim 16\,{\text{pN}}~$$

which is comparable to the force developed by Hsp70-assisted folding (see previous section) or by unassisted nascent chain folding, which develops forces in the range from 4 to 8 pN (Schlierf et al. 2007).

Proteins are equally enriched in acidic and basic residues. *Δψ* only pulls acidic residues from the cytosol into the periplasmic space, but it moves basic residues in the opposite direction. Nevertheless, the accelerating effect of PMF on translocation (Daniels et al. 1981; Yamane et al. 1987) can—although to a lower degree—also be observed for substrates enriched in basic residues (Liang et al. 2012). The decline in rate and efficiency of translocation both depended on the distribution of basic residues and their total number. Thus, the positive PMF effect on the translocation of stretches with positively charged amino acid side chains indicates that ΔpH must be involved. This conclusion is in line with the observation that the translocation rate dropped to 30% in heavy water. Such an isotope effect can only be explained by the involvement of proton transfer reactions (Springer et al. 2011). ΔpH was reported to somehow facilitate the passage of (i) bulkier substrates (Bonardi et al. 2011) or (ii) larger polypeptide segments after their release from SecA (Mitra et al. 2006). For translocation of other substrates, ΔpH does not seem to be essential (Koch und Müller 2000).

Currently it is not understood how ΔpH drives translocation. Any putative mechanism should account (i) for the lack of a net charge since acidic and basic residues are roughly equal in number for most polypeptides and (ii) for the variability of ΔpH and *Δψ* in neutralo-, acido-, and alkali-philes. *E. coli* belongs to the neutralophiles and hence, it has a moderate ΔpH of about 0.5–1.5 pH units (acidic in the periplasm) and a high *Δψ* of about − 100 to − 150 mV (negative in the cytoplasm) (Krulwich et al. 2011). Acidophiles have a larger ΔpH but a smaller *Δψ* of opposite polarity (Ingledew 1990). Alkaliphiles have a moderate ΔpH (basic in periplasm), but a large *Δψ* (negative in cytoplasm) (Sturr et al. 1994). We believe that the considerable pKa shift of titratable residues within the translocon is key for the understanding of how a universal mechanism may cope with such diversity.

### The Putative Mechanism of PMF-Driven Translocation

Both pK_a_ shifts and a pH microclimate within the translocon appear to be crucial to the PMF-driven translocation.

(i) The pK_a_ values of both basic and acidic residues shift towards neutral values within the translocation pore due to the well-known dependence of pK_a_ on the dielectric properties of the environment. It is thought to be based on minimizing the electrostatic energy penalty known as Born energy for putting a charge into a medium with low electric permittivity. The relative permittivity ε_r_ amounts to ~ 2 in the lipid bilayer core (Huang und Levitt 1977) and to ~ 3 inside proteins (Kukic et al. 2013). The low ε_r_ value agrees with the observed high ordering of water molecules inside the translocon by molecular dynamic simulations (Capponi et al. 2015). A pK_a_ shift of 3–5 pH units was reported for pH-sensitive dyes in dioxane-water mixtures upon lowering the dielectric constant ε_r_ from 80 to 10 (Fernandez und Fromherz 1977). Similarly, pK_a_ shifts of up to 5 pH units were observed for both acidic (Isom et al. 2010) and basic residues (Isom et al. 2011; Robinson et al. 2014), when transferring them into apolar proteinaceous surroundings.
(ii) pH within the translocon pore is more basic than in the cytoplasm. This is due to SecY’s moderate anionic selectivity: Since the permeability for anions is sevenfold higher than for cations (Sachelaru et al. 2017), positively charged particles must be partially excluded from the pore. Under physiological PMF this pH shift is expected to be even larger, because voltage gating of SecY (Knyazev et al. 2014) narrows the translocation pore, which in turn exacerbates electrostatic effects.

**Fig. 6:**
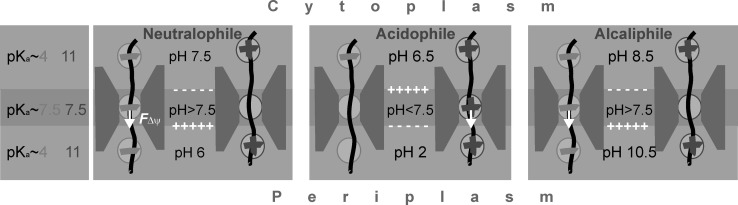
PMF-driven translocation. Both pH microclimate and pK_a_ shifts in the constriction zone of the translocon (grey), which alter the probability of being charged for both basic (blue) and acidic residues (red) to the effect that only the forward movement (towards the periplasm) of the translocating peptide chain (black line) is facilitated by the transmembrane potential *Δψ* (white arrows). Left panel: Slabs with three different dielectric permittivities are distinguished and the resulting approximate pK_a_ of titratable amino acid side chains is indicated for both acidic (red) and basic (blue) residues. Right panels: Indicates whether the titratable amino acid side chains are expected to be charged (plus or minus inside a circle) or uncharged (empty circles). ΔpH values and *Δψ* polarities are taken from literature: (Krulwich et al. 2011; Zilberstein et al. 1979; Ingledew 1990; Sturr et al. 1994), left to right. (Color figure online)

To reflect the heterogeneity of *ε* in the membrane, we divide the substrate pathway into three slabs. In the outer two (light blue on Fig. 6), we assume *ε* to be close enough to its bulk value so that the titratable residues will have bulk-like pK_a_ values. In the middle slab (green), pK_a_ will be shifted due its low *ε*.

Since the channel has an hourglass shape (Fig. 1), the middle slab is also the pore restriction site for secretory proteins. One would obviously expect channel resistivity to ion flow be greatest at this most constricted region of the pore. As a result, the electrical transmembrane field is focussed onto exactly the same spot where the shift in *ε* is largest. Local charged groups or the surface charge on protruding lipids may affect the dielectric properties of translocon’s pore, but they do not shift the constriction zone. That is, the region of steepest potential profile remains well aligned with the region in which pKa is most affected.

Consequently, we may assume the pK_a_ of basic residues (lysine and arginine) to be equal to about 11 in the outer slabs, and that of acidic residues (glutamate and aspartate) to be equal to about 4. These pK_a_ values shift to about 7.5 for both types of residues in the middle slab. pH within the two outer slabs is equal to the respective bulk values of cytoplasm and periplasm. pH of the middle slab is equal to their arithmetic mean to which the pH shift $\delta (pH)$ has to be added which arises from translocon’s anion selectivity. Based on these assumptions we arrive at pH ≈ 8 for the middle slab (Fig. 6, Neutralophile).

The pH microclimate in the constriction zone of the translocon renders the probabilities of being charged unequal for basic and acidic residues. In consequence, Δψ ensures the uni-directionality of polypeptide translocation from the cytoplasmic to periplasmic sides (Fig. 6). Specifically, the acidic residues of amino acid side chains are likely to be charged when the polypeptide traverses the constriction side of the translocon in neutralophiles (*E. coli*) and alkaliphiles (*Bacillus pseudofirmus*). Consequently, *Δψ* exerts a net electrostatic force on the peptide chain that is directed towards the periplasm (Fig. 6). In contrast, polypeptide’s basic residues are charged for acidophiles (*Acidithiobacillus ferrooxidans*). The net electrostatic force will still be directed towards the periplasm since *Δψ* has an inverted polarity in acidophiles (Fig. 6).

To prove the proposed mechanism of PMF-driven translocation (Fig. 6), precise control over both ΔpH and *Δψ* must be executed in the experimental system. This requirement rules out both IMVs and reconstituted vesicles. From that perspective, SecY reconstitution into free-spanning lipid bilayers appears most appealing—if combined with single-molecule fluorescence techniques.
